# Identifying inequities in maternal and child health through risk stratification to inform health systems strengthening in Northern Togo

**DOI:** 10.1371/journal.pone.0173445

**Published:** 2017-03-16

**Authors:** Katharine J. McCarthy, Sandra Braganza, Kevin Fiori, Christophe Gbeleou, Vivien Kpakpo, Andrew Lopez, Jennifer Schechter, Alicia Singham Goodwin, Heidi E. Jones

**Affiliations:** 1 Graduate School of Public Health and Health Policy, City University of New York, New York, United States of America; 2 Population Council, New York, New York, United States of America; 3 Department of Pediatrics, Children’s Hospital at Montefiore, Bronx, New York, United States of America; 4 Department of Family and Social Medicine, Montefiore Medical Center, Bronx, New York, United States of America; 5 Hope Through Health, Kara, Togo, New York, New York, United States of America; 6 Kozah District Health Department, Ministry of Health, Kara, Togo; London School of Economics and Political Science, UNITED KINGDOM

## Abstract

**Objective:**

In Togo, substantial progress in maternal and child health is needed to reach global development goals. To better inform clinic and community-based health services, this study identifies factors associated with maternal and child health care utilization in the Kara region of Northern Togo.

**Methods:**

We conducted a population-representative household survey of four health clinic catchment areas of 1,075 women of reproductive age in 2015. Multivariable logistic regression was used to model individual and structural factors associated with utilization of four maternal and child health services. Key outcomes were: facility-based delivery, maternal postnatal health check by a health professional within the first six weeks of birth, childhood vaccination, and receipt of malaria medication for febrile children under age five within 72 hours of symptom onset.

**Results:**

83 percent of women who gave birth in the last 2 years delivered at a health facility. In adjusted models, the strongest predictor of facility delivery in the rural catchment areas was proximity to a health center, with women living under three kilometers having 3.7 (95% CI 1.7, 7.9) times the odds of a facility birth. Only 11 percent of women received a health check by a health provider at any time in the postnatal period. Postnatal health checks were less likely for women in the poorest households and for women who resided in rural areas. Children of polygamous mothers had half the odds of receiving malaria medication for fever within 72 hours of symptom onset, while children with increased household wealth status had increased odds of childhood vaccination and receiving treatment for malaria.

**Conclusion:**

Our analysis highlights the importance of risk stratification analysis to inform the delivery and scope of maternal and child health programs needed to reach those with the least access to care.

## Introduction

The burden of poor maternal and child health outcomes in Togo has remained persistently high over the past decade, with an estimated maternal mortality ratio of 398 deaths per 100,000 live births and an under five child mortality rate of 88 deaths per 1,000 live births [1,2]. Progress in reducing these rates has been slow and variable. Togo did not achieve Millennium Development Goal (MDG) four or five, which are global targets to reduce child and maternal mortality rates by two-thirds and three-quarters their 1990 levels by 2015, respectively. To achieve these goals, Togo would have needed to reduce child mortality by an additional 25 percent and maternal mortality levels by an additional 43 percent [1].

To accelerate progress, the Togolese government has initiated multiple health sector reforms. In 2011, the government adopted a new national health policy and health development plan for 2012 to 2015. Facilitated by its engagement with the International Health Partnership (IHP+), Togo also signed a national compact to support the plan’s implementation [3]. The Togolese government has also committed to global initiatives such as the Campaign on Accelerated Reduction of Maternal Mortality in Africa (CARMMA), the main objective of which is to expand the availability of use of quality maternal health services [4]. While the health system remains largely financed through out-of-pocket expenses, as a result of these efforts government expenditure on health increased to 15 percent of the national budget in 2015. Mandatory social insurance was also introduced for public-sector workers, who comprise approximately ten percent of the population [3]. While Togo currently lacks a formal policy on free health care for children and pregnant women, the government removed user fees for specific health initiatives. In 2011, the government declared free malaria treatment for children under the age of ten [5,6]. Additionally, with support from the GAVI Alliance, Togo has provided the pentavalent vaccine at no cost through its Expanded Immunization Program (EIP) since 2008 [7].

These efforts represent substantial changes to health policy in Togo; however, they have been insufficient to achieve progress in maternal and child health. Between 2000 and 2011 the annualized rate of decline in maternal and child mortality was only 1.9 percent and 1.2 percent, respectively, leaving approximately 1,800,000 women of reproductive age and 1,033,000 children under age five at risk of poor maternal and child health outcomes [8]. With targets set by the Sustainable Development Goals calling for a global maternal mortality ratio of less than 70 deaths per 100,000 live births and the end of preventable newborn and under five child deaths, it is clear that a new approach for improving lives is needed to catalyze change [9].

The lack of anticipated improvement in maternal and child health is in part driven by vast inequities in the effective coverage of essential health interventions. While access to care is only one component of effective coverage, which is contingent on quality of care and receipt of care among those in need of services, existing evidence shows that women with fewer resources are less likely to access health services [10]. For example, attendance by a health professional at birth is 94 percent among those in the highest wealth index, while it is 28 percent among the country’s poorest [11]. Inequities also exist in terms of geographic location, delivery by a health professional exceeds 90 percent for urban populations while remains less than half for those living in rural areas (43%) [11]. In the country’s northern regions such as Kara, maternal and child health outcomes are notably poor: the under-five mortality greatly exceeds the national level at rate of 130 deaths per 1,000 live births [2]. These figures provide further evidence that those intended to benefit from health reform policies—poor and marginalized women and children, are not being engaged in care.

In addition to wide social and geographic disparities in health care utilization, Togo has tended to be overlooked by the international community. Official Development Assistance (ODA) to Togo declined from 11.9% of GDP in 1990 to 2.5% in 2003 [12]. Global public assistance also remains significantly lower in Togo than in neighboring countries: net ODA received per capita was 31.8 for Togo in 2013 while it was 50.9 for Ghana, 60.9 in Burkina Faso and 63.2 in Benin [13]. In contrast, Togo’s gross national income (GNI) per capita was nearly three times lower than that of Ghana in 2015 (US$540 and US$1,480, respectively) [14]. Inadequate funding is one element that has hampered the capacity of the national healthcare system to provide adequate care, as many countries in sub-Saharan Africa rely on international funding as a catalyst to strengthening healthcare systems [15]. For example, such funding could be used to strengthen human resources in health for which there are currently 2.7 nurse/midwife professionals per 10,000 population [16].

Since 2004, the U.S. based non-profit organization Hope through Health (HTH) has sought to increase access to essential maternal and child health services for women and children in the northern Kara region of Togo. In 2015, HTH expanded its HIV care delivery system to provide broader maternal and child healthcare services through an integrated clinic and community-based health systems strengthening model implemented in partnership with the Government of Togo. To strengthen and tailor this intervention, the objective of this study was to identify individual-level and structural factors that prevent women from accessing key maternal and child health services in the Kara region using a risk stratification approach. Data are drawn from a cross-sectional population-representative household survey, prior to intervention initiation.

We assessed factors associated with facility-based delivery, a health check for the mother by a health professional at any time in the postnatal period, childhood vaccination under age five for the pentavalent vaccine, and febrile children under age five who received antimalarial treatment. Findings not only inform the HTH program model, but also inform gaps in broader health system strengthening efforts in the region, such as adoption of WHO standards for improving quality maternal and newborn care [17]. Such information can guide adequate and efficient allocation of resources and ensure locally relevant policies meet the needs of marginalized women.

## Methods

### Ethical review

This study was reviewed and approved by the Albert Einstein College of Medicine Ethics Review Committee (#005127) and the Comité de Bioéthique pour la Recherche en Santé (#031/2014/CBRS) in Togo. Secondary analysis of the de-identified dataset was deemed exempt from City University of New York IRB review. Prior to study participation, all participants provided written informed consent. All respondents were ages 18 and older and able to consent to the study as an adult.

### Study setting

The study took place in the Kozah district of the Kara region in Togo, which covers an area of about 1,075 km^2^ with an estimated population of 225,259 [18]. Four catchment areas of public health clinics operated by the Ministry of Health were selected by the District Health Director based on the limited availability of maternal and child health services and where it was of interest to identify factors contributing to low utilization rates in the area. The four catchment areas were the urban area of Adabawere and three rural areas: Kpindi, Sarakawa and Djamdé. In 2014, the coverage rates of facility access were 54 percent in Adabawere, 23 percent in Kpindi, 15 percent in Sarakawa and 36 percent in Djamdé. The total population served by the four clinics is approximately 21,457 [18].

### Power analysis

The present analysis draws upon baseline cross-sectional data collected as part of a larger study led by HTH and the Ministry of Health to reduce child mortality over a three-year period. For the main study, a sample size of 1,500 respondents at baseline and, again, post-intervention is needed in order to detect a 75 percent reduction in the under-five childhood mortality rate between the current baseline survey and a future post-intervention survey [19]. This sample size was calculated based on the following assumptions: baseline under-five child mortality rate of 88 deaths per 1,000 live births, design effect of 1.5, total fertility rate of 4.8 [2], a 20% non-response rate, with a 95% confidence level and a 5% margin of error in a population of 21,457 [18].

For the present secondary analysis, we anticipated adequate power to examine individual and structural-level differences in the four maternal and child health care outcomes of interest with the following assumptions: 50% prevalence for all indicators (given the potential for some indicators to be high prevalence, while others may be low prevalence), 80% power, and an alpha of 0.05. We considered a difference of at least twenty-percentage points between exposure groups to be programmatically meaningful. Under these specifications, a sample size of 206 is required for adequate power.

### Sampling methodology

We conducted a cross-sectional household survey among women between the ages of 18 to 49 between January 19 and February 24, 2015. A probability-based cluster sampling strategy was applied in two phases. First, the Kara region was stratified by four main catchment areas around the selected health centers (Adabewere, Kpindi, Sarakawa and Djamdé). Next, using geographic information system (GIS) technology, each catchment area was divided into 15 clusters of approximately equivalent size according to population density reports from Togo’s 2010 Census to comprise the primary sampling unit [18]. Within each cluster, data collectors conducted door-to-door recruitment. Starting points were chosen at random by dropping a pen on the map to determine a starting location, and spinning the pen to determine the starting direction to head in to avoid systematic bias in the types of households visited by data collectors.

Households were considered eligible if at least one adult female resided within and was present at the time of recruitment. If more than one eligible female resided in the household, the respondent was randomly selected using a KISH table [20]. Based on 2010 census data and the understanding that not all households approached would have eligible females at home during fielding, we estimated we would need to approach 50% of households in each catchment area to achieve a sample size of 1,500 respondents. During data collection, however, field staff noted that the population exceeded census estimates in the urban area of Adabawere and thus 25% of households were approached in this area. Population-based weights were calculated to adjust for non-equal probability of selection (i.e., 50% coverage in each rural site and 25% coverage in Adabewere) and to adjust for participant non-response [21].

Data collectors were local individuals with at least some post-secondary education who were also fluent in French and Kabiyé. Many of the interviewers had previously worked on the 2013 Demographic and Health Survey and/or the 2010 Togo Census. All data collectors received a detailed three-day training led by HTH researchers on the ethical procedures for conducting research with human subjects, the procedures for data collection and obtaining informed consent, as well as the sampling strategy and administration of the interview questionnaire. Data collectors recorded participant responses using pen and paper questionnaires, with close supervision by the research team and HTH staff to ensure data quality and accuracy.

### Household questionnaire and measures

Survey questions related to household demographics, maternal and newborn health, family planning, and child disease and treatment were drawn from modules from the French-language versions of the 2013 Demographic and Health Surveys (DHS) and 2010 Multiple Indicator Cluster Survey (MICS) in Togo, with a few minor adaptations. Following the maternal and newborn health module, women responded to questions related to receipt of antenatal, labor and delivery and postnatal care for live births within the two years preceding the survey. Women also responded to questions on birth history from the last 10 years, children currently living with them, and child disease and treatment status (e.g., fever, cough and diarrhea). To reflect a more recent child cohort, questions related to child disease and treatment refer to a woman’s most recent birth among all children born in the past 10 years. This slightly differs from the DHS, which represents the most recent birth among all prior births.

### Outcomes

#### Maternal health utilization

A critical intervention in reducing maternal mortality is increasing women’s timely access to emergency obstetric services in order to manage life-threatening complications that occur during labor and delivery or postnatal periods. Given challenges in measuring the receipt of quality intrapartum care from women by self-report in a survey [22–24], we assessed facility-based delivery by asking women who had a live birth in the two years preceding the survey to report where their most recent birth occurred. Facility-based delivery is a widely used indicator of health care access, routinely tracked in health initiatives such as the Millennium Development Goals (MDGs) and Sustainable Development Goals (SDGs) to measure one component of effective intervention coverage. Responses were dichotomized into facility (e.g., public or private sector health post, community health center, hospital or clinic) or home births. Women with a live birth in the past two years were also asked about whether they received a postnatal health care check by a health professional (i.e., a doctor, clinical assistant, nurse-midwife or auxiliary midwife) at any time in the first 42 days following delivery. The standard of care for postnatal care is a health check within the first two days of birth [25]. However, less than two percent of women (N = 7 cases) reported a health check within this period. Given that nearly 2 in 5 maternal deaths occur in the postnatal period [26], we defined this outcome to include postnatal health care checks by a health professional at any time in the postnatal period (i.e., within the first 42 days of delivery). Current WHO recommendations related to postnatal care do not stipulate that health checks take place within a facility setting [27]. We also present these results by the type of health professional seen.

#### Child health utilization

We calculated the proportion of children who: (1) ever received the third dose of the pentavalent vaccine (or DTCoq3) which protects against diphtheria, pertussis, tetanus, hepatitis B and haemophilis influenzae type B, and (2) the proportion of children in the household under age five reported to have been febrile in the two weeks preceding data collection, who received an effective antimalarial treatment (ACT, arthemeter, arthesunate or artmefloquite or quinine) within 72 hours of symptom onset. These indicators of health service coverage were chosen to reflect key initiatives in child health in Togo, such as no-cost malaria treatment for children [5,6] and child vaccination through the Expanded Immunization Program (EIP) [7]. For child vaccination, cases were restricted to the youngest child born to women in the last ten years. Cases where vaccination status was unable to be determined (14.6%) were excluded from multivariable analysis for this outcome. In instances where more than one febrile child among women was reported in the past two weeks, the first reported case was analyzed.

### Risk factors

#### Individual-level attributes

Personal characteristics of women examined include age, highest educational attainment, marital status (i.e., currently married or living together with a man versus not currently in a union), polygamous versus monogamous marriage, prior parity and ethnic group.

#### Structural factors

We considered factors that affected the logistics of accessing care to include household wealth quartile, health insurance status, facility-proximity (distance in km), and site of residence. Household wealth status was measured using household assets on a scale ranging from 0 to 11 with one point given for each of the following household items (electricity, running water inside compound, corrugated tin roof, radio, television, mobile phone, refrigerator, motorbike, bicycle, car, or gas appliance stove). We used wealth quartiles as an index of household socioeconomic status measure in subsequent analysis. Health insurance was measured as women’s self-reported coverage status.

Facility proximity was assessed as the distance to the nearest health facility (either a health post, community health center, clinic or hospital) within each cluster (less than 3km, between 3-5km, or greater than 5km by distance or by road). Distance to the nearest health facility was measured using global positioning systems (GPS) technology available through Google Maps to measure a three and five-kilometer radius from each health facility. Each of the 15 clusters within the four catchment areas were categorized “as the crow flies” according to whether more than 50% of households fell within the three-kilometer radius, within the five-kilometer radius, or exceeded this distance. We also considered differences in maternal and child health utilization across the four sites of residence (catchment areas of Adabewere, Djamdé, Sarakawa or Kpindi). Urban versus rural status was assessed by household residence in Adabewere, the only urban area, as compared to the three rural sites of Djamdé, Sarakawa and Kpindi.

### Analysis

We present bivariate and multivariable logistic regression models that assessed the above risk factors for the four outcome measures for maternal and child health service use: delivery in a health facility, a postnatal health check for the mother by a health professional, receipt of the pentavalent vaccine for children under age 5, and febrile children under age five who received antimalarial treatment within 3 days of fever onset. We accounted for clustering among observations within the same primary sampling unit and weighted results to population estimates by using the “svy” function for complex survey data in Stata 14 (StataCorp, College Station, TX, USA). Descriptive statistics (raw sample sizes and population-weighted percentages) are reported for each health outcome. Given that the sample of women who reported on maternal (women with births in the last two years) and child (women with births in the last ten years) health outcomes differed, we disaggregate participant characteristics for each subgroup. We compared women’s sociodemographic characteristics and maternal and child health care utilization using chi-squared tests for association reporting within group prevalence estimates and multivariable logistic regression reporting adjusted odds ratios. Due to limited sample sizes of distinct ethnic groups, bivariate data on ethnicity are not presented in the data tables and ethnicity was not included in the final multivariable analysis. Multivariable models were developed in an iterative process, including variables significantly associated with the outcome of interest at the bivariate level and for which there was sufficient sample size for robust analysis (>5% prevalence within the total and rural specific strata). Pairwise missing data were excluded. Final models were selected to maximize goodness of fit indices using a backwards elimination strategy to identify variables most predictive of the outcome of interest, while adjusting for complex sampling and applying population-based weights.

To account for potential time trends in the likelihood of childhood vaccination among women’s youngest child born in the past ten years, we adjusted for child age in the multivariable model of childhood vaccination status. As area of residence corresponded with perfect prediction of facility-based delivery, we present multivariable findings stratified by urban and rural residence.

## Results

### Sample descriptive characteristics

Of the target 1,922 households, a total of 1,330 women ages 18 to 49 agreed to participate. Twenty-eight percent of households had no adult female present in the home at the time of the survey and were excluded from the sample (S1 Table). Less than five percent of the sample refused to participate or were unable to complete the survey. Of eligible respondents, a total of 1,075 women reported a live birth in the 10 years preceding the survey. Of these women, 45 percent had a live birth in the past two years.

Women’s use of maternal and child health services varied depending on the intervention (Table 1). More than three-quarters (83%) of women with a birth in the two years preceding the survey reported that their most recent delivery took place in a health facility. Health posts were the most common type of facility (49%), followed by hospitals (20%), other public/private facilities (11%) and health centers (3%). Nearly one-fifth of births took place in the home (17%). Only 11 percent of women received a postnatal health check by a health professional (i.e., doctor, nurse/midwife, clinical assistant or auxiliary midwife) at any time in the first six weeks of birth, while 26% reported receiving a check by a non-health professional (matrone, traditional birth attendant, community health worker, or friend/relative). Among women with a birth in the preceding two years, less than two percent of women received a health visit by a provider in the first two days of birth and only ten percent of women were visited within the first 14 days of birth. Women were more likely to receive a health check by a health professional sooner than another type of individual. Twenty one percent of women who received a health check by a health professional received the visit within the first two days of birth compared to four percent of women visited by a lay person. By 14 days of birth, 91% of women visited by a health professional received a check compared to 31% of women visited by a non-professional. Coverage of childhood vaccination was 83 percent. Excluding cases that were unable to be determined (i.e., the mother could not recall and no record could be found), childhood vaccination prevalence was 94 percent. Among children under the age of five, the fever prevalence was 39 percent in the past two weeks. Of these cases, 42 percent of children received appropriate medication for treatment of malaria within 72 hours of onset of symptoms.

**Table 1:** Percentage distribution of maternal and child health service utilization, HTH population-representative household survey of 4 catchment areas in Kara region, Togo, 2015.

	Percentage, % (Weighted)	95% CI (Weighted)	N (Unweighted), Total = 1075
**Women with live birth in last 2 years**	45.4	(41.4, 49.5)	**490**
**Type of facility**			
Home	17.1	(12.7, 22.5)	110
Facility^a^	82.9	(77.5, 87.4)	378
Total			**490**
**Postnatal health check for mother (within first 42 days of birth, by anyone)**			
**No**	**60.9**	**(50.7, 70.3)**	**302**
**Yes**	**36.9**	**(27.4, 47.5)**	**177**
***Check by health professional***			
Doctor	0.6	(0.2, 1.8)	3
Clinical assistant	0.4	(0.1, 1.7)	2
Nurse midwife/ auxiliary midwife	9.9	(5.1, 18.2)	38
***Check by non-health professional***^c^			
Matrone	20.3	(15.5, 25.5)	105
Traditional birth attendant	2.2	(0.9, 5.2)	7
Community health worker	2.5	(1.2, 4.9)	13
Friend or relative	1.4	(0.1, 2.8)	9
**Missing**	**2.2**	**(1.0, 4.8)**	**11**
Total			**490**
**Youngest child among mothers with births in past 10 years**			
**Child vaccination**			
No	5.0	(3.2, 7.6)	48
Yes	83.0	(77.8, 87.1)	635
Unable to determine	12.2	(8.7, 16.6)	115
Total			**798**
**Children living under age 5**			
**Fever prevalence (last 2 weeks, children <age 5)**			
No	61.4	(54.9, 67.6)	510
Yes	38.6	(32.4, 45.1)	334
Total			**844**
**(Of children <age 5 with fever in last 2 weeks) Antimalarial drugs given in <3 days of fever onset**^b^			
No	57.8	49.6, 65.6	190
Yes	42.2	34.4, 50.4	144
Total			**334**

^a^ Facility includes hospital, health post, health center and other private facilities.

^b^Antimalarial drugs include: ACT, arthemeter, arthesunate, artmefloquite or quinine.

^c^ Matrone refers to a non-professional nurse.

### Access to facility-based delivery and postnatal care

Table 2 presents the percent distribution of women’s access to maternal health services by individual-level attributes and structural factors. Live births to women in the past two years most often occurred among those ages 25 to 34 years old (55%), who had secondary or higher education (47%), and were in the middle-bottom wealth quartile (38%). More than one-quarter of women were in a polygamous marriage (27%). Few women had health insurance (12%) and the majority lived within three kilometers of the nearest health facility (67%). Within the three rural sites, however, only 36% of women lived within three kilometers of the nearest health facility.

**Table 2:** Percent distribution of women by sociodemographic characteristics and access to maternal health care, women with live births in the last 2 years, HTH population-representative household survey of 4 catchment areas in Kara region, Togo, 2015.

Women with Live Birth in Last 2 Years						
	Total		Facility Delivery		PNC Check by Health Professional^a^	
	N = 490 (Unweighted)	Weighted Percent (%)	Weighted Percent (%)	P, value	Weighted Percent (%)	P-value
**Age Group**				0.499		0.547
18–24	**160**	**31.5**	81.9		12.5	
25–34	**262**	**55.3**	84.5		11.0	
35–49	**68**	**13.1**	79.3		7.5	
**Education Level**				<0.001*		0.089
None	**93**	**15.2**	61.0		5.5	
Primary	**202**	**37.4**	77.3		7.0	
Secondary+	**194**	**47.4**	94.8		16.0	
**Marital Status**				0.951		0.533
Single	**30**	**5.5**	83.2		14.1	
Partnered	**460**	**94.5**	83.0		10.9	
**Polygamous Marriage**				<0.001*		
No	**313**	**72.3**	85.9		12.8	
Yes	**142**	**27.7**	74.5		5.1	
**Prior Parity**				0.006*		0.472
One Birth	**140**	**34.1**	92.4		14.3	
Two Births	**156**	**37.0**	82.2		11.3	
Three+ Births	**127**	**29.0**	83.1		10.3	
**Wealth Quartile**				<0.001*		0.008*
Lowest	**130**	**22.0**	63.9		2.9	
Middle Bottom	**207**	**37.5**	81.4		14.1	
Middle Upper	**68**	**16.8**	91.8		9.6	
Highest	**84**	**23.7**	97.0		22.5	
**Insurance Status**				0.011*		0.004*
Uninsured	**447**	**88.2**	80.8		8.3	
Insured	**41**	**11.8**	100.0		31.7	
**Facility Distance**				<0.001*		0.084
>5km	**156**	**23.1**	55.6		6.6	
3-5km	**58**	**10.1**	64.0		4.3	
<3km	**276**	**66.8**	95.3		13.6	
**Site**				<0.001*		0.024*
Adabewere	**162**	**48.6**	100.0		17.5	
Kpindi	**98**	**17.2**	72.5		1.9	
Sarakawa	**111**	**17.2**	53.6		4.7	
Djamdé	**119**	**16.9**	74.7		8.7	
**Residence**				<0.001*		0.004*
Urban	**162**	**48.6**	100.0		17.5	
Rural	**328**	**51.4**	66.9		5.0	

*Statistically significant difference global Chi-Square test of association at p<0.05, adjusting for complex sampling and weighted to population-level distribution.

^a^ Postnatal health check is defined as a health check by a health professional (doctor, clinical assistant, nurse/midwife or auxiliary midwife) at any time during the first 42 days following birth.

In bivariate analyses, higher education, not being in a polygamous marriage, higher wealth quartile, facility proximity, and residing in an urban area increased the likelihood of facility-delivery (p<0.001, respectively). All women who resided in the urban area (Adabewere) delivered in a health facility, compared to 67% of women who resided in rural areas. There was no relationship between marital status and facility delivery.

In terms of accessing a postnatal maternal health check by a health professional, a higher prevalence of postnatal care was found among women residing in the households with higher wealth status: 23 percent of mothers in the highest wealth quartile received a postnatal health check from a health professional compared to 3% of women in the lowest quartile (p = 0.008). The prevalence of postnatal care was four times as high among women with insurance compared to those without (32% vs. 8%; p = 0.004) and double among women who resided within three kilometers of the closest facility compared to more than five kilometers distance (14% vs. 7%, marginal significance at p = 0.084). Differences in geographic area were noted between study sites, with the highest prevalence in Adabewere (18%), followed by Djamdé (9%), Kipindi and Sarakawa (5% and 2%), respectively (p = 0.024).

### Childhood vaccination

Table 3 presents data on women’s access to child health services stratified by individual and structural-level attributes of mothers who gave birth in the ten years preceding the survey. Other than age, the characteristics of women who gave birth in the past ten years were similar to women who gave birth in the two years preceding the survey.

**Table 3:** Percent distribution of women by sociodemographic characteristics and access to child health care, women with live births in the past 10 years, HTH population-representative household survey of 4 catchment areas in Kara region, Togo, 2015.

Women with Live Birth in Past 10 years			Childhood Vaccination		Antimalarial Drugs Given to Febrile Children in <3 days of Fever Onset^a^	
	Total N (Unweighted)	Weighted Percent (%)	Youngest Living Child Born in Last 10 Years		Children Under Age 5 with Fever in Past 2 weeks	
			Weighted Percent (%)	P-value	Weighted Percent (%)	P-value
**Age Group**				0.026		0.265
18–24	**233**	**21.1**	93.0		43.0	
25–34	**520**	**50**	92.6		38.5	
35–49	**322**	**28.9**	97.6		50.3	
**Education Level**				0.244		0.032*
None	**235**	**17.4**	92.7		28.7	
Primary	**477**	**41.5**	92.6		42.5	
Secondary+	**359**	**41.1**	96.8		48.6	
**Marital Status**				0.239		0.167
Single	**104**	**9.7**	98.1		54.0	
Partnered	**960**	**90.3**	94.0		41.1	
**Polygamous Marriage**				0.450		0.014*
No	**397**	**65.3**	94.5		45.9	
Yes	**211**	**34.7**	93.0		26.9	
**Prior Parity**				0.115		0.213
One Birth	**257**	**40.7**	95.8		44.0	
Two Births	**251**	**39.8**	95.1		49.0	
Three+ Births	**123**	**19.5**	90.3		36.6	
**Wealth Quartile**				0.022*		0.028*
Lowest	**307**	**23.7**	88.3		27	
Middle Bottom	**442**	**38**	94.2		44.5	
Middle Upper	**151**	**16**	97.3		51.62	
Highest	**175**	**22.3**	98.2		51.23	
**Insurance Status**				0.093		0.619
Uninsured	**976**	**88**	93.7		42.6	
Insured	**93**	**12**	98.6		34.4	
**Facility Distance**				0.003*		0.541
>5km	**328**	**21.5**	89.1		34.4	
3-5km	**119**	**9.3**	85.5		48.2	
<3km	**628**	**69.2**	96.9		43.7	
**Site**				0.003*		0.077
Adabewere	**369**	**49.7**	96.9		47.8	
Kpindi	**209**	**16.9**	97.2		21.2	
Sarakawa	**243**	**16.7**	94.4		40.3	
Djamdé	**254**	**16.8**	92.8		57.9	
**Residence**				0.036*		0.213
Urban	**369**	**49.7**	96.9		47.8	
Rural	**696**	**50.3**	91.4		37.9	

*Statistically significant difference global Chi-Square test of association at p<0.05, adjusting for complex sampling and weighted to population-level distribution.

^a^ Antimalarial drugs include: ACT, arthemeter, arthesunate, artmefloquite or quinine.

A higher prevalence of child vaccination was observed among children whose mother was older than age 35 (98% of women ages 35–49 vaccinated her youngest child) compared to younger mothers (93% of women ages 18–24 or 25–34, respectively) (p = 0.026). The likelihood of vaccination among children also increased with higher household wealth quartile (98% among women in the highest quartile compared to 88% of those in the lowest), facility-proximity (97% of those who resided within three kilometers of the closest facility compared to <90% of those who lived farther than three kilometers distance) and among children with households in urban (97%) vs. rural (91%) areas (p<0.05, respectively).

### Malaria treatment among febrile children

In terms of malaria treatment for febrile children, nearly half (49%) of children with mothers who had secondary or higher education received treatment within the first 72 hours of symptom onset compared to 29% of those whose mothers had no education (p = 0.032). Higher treatment rates were also observed among children whose mothers had higher household wealth: 51% of children under five who resided in the highest wealth quartile received antimalarial treatment for fever compared to 27% of those in the lowest quartile (p = 0.028). Children born to mothers in polygamous marriages were less likely to receive treatment (27%) compared to those in monogamous marriages (46%) (p = 0.014). Marginal differences were observed with respect to area of residence, with the highest percentage of treated children residing in Djamdé (58%) and lowest in Kpindi (21%) (p = 0.077).

### Multivariable analysis of factors influencing use of maternal and child health services

Tables 4 and 5 present the adjusted association of individual demographic characteristics and structural factors on the ability to access maternal and child health services using multivariable logistic regression.

**Table 4:** Multivariable logistic regression models of predictors of use of maternal and child health care, HTH population-representative household survey of 4 catchment areas in Kara region, Togo, 2015.

	Women's most recent live birth in 2 years preceding survey			Women’s most recent live birth in 10 years preceding survey		Children under 5 with fever in past 2 weeks	
	(1)			(2)		(3)	
	PNC Health Check by Health Professional^a^			Childhood Vaccination		Antimalaria Drugs Given to Febrile Children in <3 Days of Fever Onset^b^ (Children <Age 5, with Fever in Last 2 Weeks)	
	Adj. OR	95%CI		Adj. OR	95%CI	Adj. OR	95%CI
**Education Level**							
None	1.0			1.0		1.0	
Primary	0.92	(0.16, 5.21)		0.47	(0.13, 1.70)	1.78	(0.80, 3.95)
Secondary+	1.16	(0.27, 5.07)		0.84	(0.19, 3.68)	1.99	(0.78, 5.06)
**Polygamous Marriage** (Yes v. No)	0.55	(0.19, 1.64		NA		0.50*	(0.26, 0.97)
**Prior Parity**							
One Birth	1.0			1.0		NA	
Two Births	0.82	(0.46, 1.49)		1.04	(0.41, 2.62)		
Three+ Births	0.82	(0.38, 1.75)		0.85	(0.39, 1.87)		
**Wealth Quartile**							
Lowest	1.0			1.0		1.0	
Middle Bottom	6.14**	(1.66, 22.70)		2.52**	(1.27, 4.99)	2.23**	(1.25, 3.99)
Middle Upper	3.66*	(1.32, 10.16)		4.55*	(1.36, 15.26)	4.02**	(1.62, 9.98)
Highest	5.93**	(1.91, 18.37)		6.97	(0.95, 51.26)	2.74	(0.96, 7.84)
**Facility Distance**							
Far (>5km)	1.0			1.0		1.0	
Close (3-5km)	0.49		(0.06, 4.34)	1.30	(0.58, 2.92)	2.06	(0.62, 6.86)
Closest (<3km)	0.59		(0.13, 2.71)	2.33	(0.57, 9.48)	1.31	(0.41, 4.15)
**Site**							
Adabewere	1.0			1.0		1.0	
Kpindi	0.15**		(0.03, 0.88)	3.44	(0.54, 21.87)	0.48	(0.09, 2.66)
Sarakawa	0.49		(0.07, 3.44)	0.94	(0.15, 6.00)	1.41	(0.31, 6.48)
Djamdé	0.51		(0.09, 2.80)	1.68	(0.39, 7.25)	3.14*	(1.02, 9.70)
**Child Age** (Years)	NA			1.52**	(1.17, 1.98)	NA	
Observations	387			614		300	

Statistically significant at ** p<0.01 and * p<0.05.

^a^ Postnatal health check is defined as a health check by a health professional (doctor, clinical assistant, nurse/midwife or auxiliary midwife) at any time during the first 42 days following birth.

^b^ Antimalarial drugs include: ACT, arthemeter, arthesunate, artmefloquite or quinine.

**Table 5:** Multivariable logistic regression models of predictors of use of maternal and child health care in rural sites only, HTH population-representative household survey of 3 rural catchment areas in Kara region, Togo, 2015.

	Women's most recent live birth in 2 years preceding survey				Women’s most recent live birth in 10 years preceding survey		Children under 5 with fever in past 2 weeks	
	(1)		(2)		(3)		(4)	
	Facility Birth, Rural		PNC Health Check^a^, Rural		Childhood Vaccination, Rural		Antimalarial Drugs Given to Febrile Children in <3days of Fever Onset^b^, (Rural Children < Age 5, with Fever in Last 2 Weeks)	
	Adj. OR	95%CI	Adj. OR	95%CI	Adj. OR	95%CI	Adj. OR	95%CI
**Education Level**								
None	1.0		NA		1.0		1.0	
Primary	0.96	(0.47, 1.97)			0.62	(0.16, 2.35)	1.56	(0.65, 3.73)
Secondary+	2.12	(0.84, 5.35)			1.15	(0.19, 6.95)	2.80	(0.94, 8.38)
**Polygamous Marriage** (Yes v. No)	0.88	(0.53, 1.44)	NA		NA		0.55	(0.27, 1.14)
**Prior Parity**								
One Birth	NA		1.0		1.0		NA	
Two Births			0.96	(0.23, 4.11)	2.4	(0.73, 6.29)		
Three+ Births			0.59	(0.12, 2.83)	1.00	(0.40, 2.50)		
**Wealth Quartile**								
Lowest	1.0		1.0		1.0		1.0	
Middle Bottom	1.53	(0.84, 2.76)	2.71	(0.85, 8.60)	2.25*	(1.03, 4.93)	1.86*	(1.10, 3.17)
Middle Upper	2.19	(0.75, 6.42)	3.26	(0.74, 14.41)	7.80	(0.80, 75.78)	3.17*	(1.06, 9.43)
Highest	1.31	(0.26, 6.52)	-		-		0.38	(0.03, 4.33)
**Facility Distance**								
Far (>5km)	1.0		NA		NA		NA	
Close (3-5km)	2.07*	(1.09, 3.96)						
Closest (<3km)	3.69**	(1.72, 7.92)						
**Site**								
Kpindi	1.0		1.0		1.0		1.0	
Sarakawa	0.58	(0.26, 1.27)	2.94	(0.43, 20.29)	0.22	(0.04, 1.29)	3.76	(0.72, 19.54)
Djamde	1.68	(0.66, 4.30)	4.38	(0.71, 26.78)	0.43	(0.09, 2.90)	6.88**	(1.74, 27.18)
**Child Age** (Years)	NA		NA		1.56**	(1.16, 2.08)	NA	
Observations	303		255		360		208	

Statistically significant at ** p<0.01 and * p<0.05.

^a^ Postnatal health check is defined as a health check by a health professional (doctor, clinical assistant, nurse/midwife or auxiliary midwife) at any time during the first 42 days following birth.

^b^Antimalarial drugs include: ACT, arthemeter, arthesunate, artmefloquite or quinine.

After adjusting for other variables in the model, the only significant factor that predicted facility-based delivery was facility proximity. The adjusted analysis for facility-based delivery is restricted to women who reside in rural areas only, as all women who resided in the urban area (Adabewere) reported that their most recent delivery took place in a health facility (Table 5). Among women who resided in rural areas, those who resided between three and five kilometers of the nearest facility had twice the odds (adjusted odds ratio, aOR 2.1, 95% CI 1.1, 4.0) of delivering in a facility than those who lived more than five kilometers distance. Those that resided within three kilometers of the nearest facility had nearly four times the odds of a facility delivery than those living more than five kilometers (aOR 3.7, 95% CI 1.7, 7.9), adjusting for all other variables in the model.

Factors predictive of a postnatal health check for mothers by a health professional differed from that of facility delivery. In the adjusted analysis women with higher household wealth status had higher odds of a postnatal health visit compared to women in the poorest households. Specifically, women in the highest wealth quartile had nearly six times the odds of a postnatal health check by a health provider relative to women in the lowest wealth quartile (aOR: 5.9; 95%CI 1.9, 18.4) Women who lived in the rural area of Kpindi had greatly reduced odds of a postnatal visit compared to those who resided in the urban area of Adabewere (aOR 0.15; 95% CI 0.03, 0.88). In the rural stratum-specific model there were no differences in access to a postnatal visit by any individual or structural characteristics.

In the adjusted analysis of factors associated with access to child health services, household wealth index was a significant predictor of both childhood vaccination and malaria treatment for febrile children in both the full sample and the rural strata-specific models. For both outcomes, however, the magnitude of effect was somewhat attenuated in the rural stratum-specific model. Adjusted analysis showed a trend for increasing childhood vaccination with time. For each additional year, the odds of vaccination increased by 1.4 and 1.6 times in the full and rural-specific samples, respectively (p<0.05).

For receipt of malaria medication for febrile children, adjusted analysis showed that children born to mothers in polygamous marriages had half the odds of receiving malaria treatment than children born to mothers in monogamous marriages in the full sample (aOR 0.50, 95% CI 0.26, 0.97). This variable was marginally significant in the rural sample (aOR: 0.55, 95% CI 0.27, 1.17). Geographic area of residence was an additional significant predictor of malaria treatment for febrile children. Children whose mothers resided in Djamdé were more than three times as likely to access malaria medication for their febrile child as compared to those who resided in Adabewere (aOR 3.14, 95% CI 1.02, 9.70) in the full sample. In the rural-specific model, children whose mothers resided in Djamdé had nearly seven times the odds of treatment as those who resided in Kpindi in the rural sample (aOR 6.88, 95% CI 1.74, 27.18).

## Discussion

Timely access to appropriate care can reduce maternal and child mortality, which remain persistently high in Northern Togo. Population averages can hide important differences in the distribution of health determinants—even among high prevalence interventions (e.g., childhood vaccination and facility delivery), leading to health inequities. In order to improve access to lifesaving interventions, health systems strengthening interventions to systematically monitor and address differences in access to care are needed. Our findings illustrate marked differences in access and coverage of essential maternal and child health interventions among subgroups of women in Northern Togo.

Our results highlight the need to address geographic barriers to facility-based delivery in rural areas of the Kara region. Results from the multivariable analyses reveal that women who reside within three kilometers of the nearest health facility are almost four times as likely to deliver in a facility relative to women who live more than five kilometers distance. Most births (49%) occurred in a health center, the most common type of facility in the catchment areas, while comparatively few women delivered in a hospital (20%) or health center (3%) which were considerably further distance (no hospitals or health centers were located within the catchment areas). Furthermore, we found that all women who reported home delivery (17% of births in the last two years) resided in a rural area. The lowest prevalence of facility delivery was in Sarakawa (54%), the same region where less than five percent of all mothers received a postnatal health visit by a health professional at any point in the first six weeks of birth. These findings are consistent with those of the 2013–14 DHS in Togo, which found that 59 percent of postpartum women nationally received no postnatal care [11]. The overlay of these geographic inequalities underscore the need for targeted outreach activities and further research to identify the mechanisms which prevent women from seeking and accessing care.

Results from our risk stratification analysis also highlight the need for active case finding among women in the immediate postnatal period and to identify and treat febrile children, preferably within 24 hours of symptom onset. A postnatal health check for mothers is particularly crucial as more maternal deaths (36%) occur in time period following the first 24 hours of birth and within six weeks of delivery than any other phase of pregnancy [28]. Our bivariate findings showed the lowest percentage of women who accessed maternal and child health services were those who resided farthest from health facilities. Given the current state of the healthcare system in Togo, which is characterized by extremely low patient to provider ratios, engaging postnatal and febrile children in care may be best facilitated by ambulatory health workers who may be able to screen women and children for danger signs and symptoms. There is growing support for interventions that utilize task shifting or “task sharing” with trained lay workers who are supervised by specialists in low and middle-income country settings. For example, studies in India and Zimbabwe have found the use of lay workers to be an effective approach in addressing common mental health disorders such as depression [29,30]. A randomized controlled trial is also currently underway to evaluate a task sharing intervention to address perinatal and postpartum depression among women in South Africa [28]. That facility proximity did not reach statistical significance for these outcomes in adjusted models may be due to limitations of sample size for high or low prevalence outcomes and lack of sufficient statistical power.

In addition to geographic disparities in access to care, our results identify important structural differences by household wealth status. The likelihood of childhood vaccination and receipt of antimalarial medication among febrile children under the age of five both approximately doubled from the lowest to low-middle wealth quartile. The difference was further augmented between the lowest and middle-upper quartile. Our bivariate results provide some evidence to suggest that health insurance coverage is an effective means to address inequalities by socioeconomic status. For example, the prevalence of facility-delivery and a postnatal health visit both increased by 23 percentage points among women with health insurance coverage. However, the small sample of insured women limited the ability to include this variable in the adjusted analysis. Despite this, our findings lend support to emerging initiatives such as the call for universal health coverage in low and middle-income countries, [31] as well as the expansion of the public health insurance scheme in Togo, which is currently provided to public service workers. We note that health efforts to increase facility utilization must be coupled with health system strengthening to adequately equip the facilities with needed equipment and supplies and to ensure adequate staffing and provider training.

Finally, our analysis found that structural level inequities tended to predict health utilization more than individual-level attributes, with the exception of the presence of co-wives (i.e., a polygamous marriage). One potential explanation is that increased family size (due to co-wives and their children) lowers household resources, thus reducing structural assets. These results further support the importance of economic resources in attaining health care and are consistent with those documented nationally [2]. For example, nationally 96 percent of women in the highest wealth quintile delivery in a health facility, compared to 43 percent of those in the lowest [11]. At the national level, women in rural areas are also more likely to deliver at home (39%) compared to those in urban areas (6%).

This study has several limitations. One limitation was that in our sampling strategy, households without an eligible participant were not re-contacted. It is possible that women who were not present in the home during recruitment hours systematically differed from those included in the study, which could bias our study findings. Additionally, our data relied on women’s recall of aspects of intrapartum, postnatal and child health care. While we used the same survey questions as those used in the most recent Togo DHS and MICS, recent studies that have sought to validate women’s reporting accuracy on such elements of care have generally found that few indicators are recalled with accuracy, although some indicators may be suitable to assess population-based intervention coverage [22,24,32,33]. We note the inherent limitations in using indicators of health care access as a proxy for receipt of essential services [34]. For example, some women interpreted a visit by someone to check on their health following delivery to include family members and relatives, which artificially augments the number of women who received a postnatal health check. To limit the potential for this bias, we used the indicator of postnatal health check by a health professional. We also clarify that access to a facility or health provider does not equate with quality of care, as access to a health facility for delivery also necessitates the presence of a trained provider, necessary supplies and equipment, and that the required content of care is received [17]. Future analyses of intervention coverage should be corroborated with data from other sources related to facility-level readiness to effectively manage obstetric complications. Our assessment of receipt of antimalarial medication for febrile children relies on the inherent assumption that all febrile children require treatment for malaria, which may be inaccurate. Additionally, for the outcomes of a postnatal health check for the mother and febrile child treatment for malaria, there were too few cases where the intervention was received within the recommended time frame (i.e., a postnatal health check within 2 days for the mothers and malaria medication within 24 hours of fever onset for children). Results from this study therefore identify women and children who received any health care, rather than the standard of care. Finally, the intention of this study was to identify commonalities in the types of barriers to salient health interventions in the Kara region to inform policy and programmatic services. We acknowledge that other interventions related to the use of family planning services and antenatal care are also key in improving maternal and child health outcomes and warrant investigation.

Despite these limitations, our findings demonstrate the need for local empirical evidence to be incorporated in the design and delivery of maternal and child health services. These efforts have particular applied relevance in Northern Togo where rates of maternal and child mortality remain high and progress has been slow. While recent guidelines such as the WHO standards to improve the quality of maternal and newborn care are necessary and promising [17], our findings make evident that equally paramount are strategies to track and understand the varied dynamics which prevent women from being engaged in facility-based care. Risk stratification analysis can aid in ensuring global policy initiatives are not disconnected from the needs of local communities, leading to a gap between empirical evidence and proposed solutions. For example, study findings are now being used to prioritize outreach efforts to low health coverage catchment areas and lend support to an integrated clinic and community-based outreach approach currently being piloted by the organization Hope for Health in partnership with the Togolese Ministry of Health. In parallel to these efforts, additional expansions to both structural access and policy (e.g., postnatal care) are warranted to improve maternal and newborn health utilization in the Kara region.

## Conclusion

Our findings highlight the need to strengthen the healthcare delivery system in order to deliver maternal and child health services to rural and poor women in Northern Togo. Recent health sector reforms demonstrate Togo’s willingness to strengthen the national healthcare delivery system. To address economic and geographic barriers to care among marginalized women promising strategies include ambulatory outreach efforts that use task sharing among community health workers and the expansion of health insurance coverage. We argue for parallel investments to strengthen health systems. Global partnerships and strong monitoring and evaluation systems can inform these efforts to achieve sustainable progress in improving maternal and child health.

## Supporting information

S1 Table Participation enrollment by study site, HTH population-representative household survey of 4 catchment areas in Kara region, Togo, 2015. (DOCX)
