# Supplementary material for: Identifying inequities in maternal and child health through risk stratification to inform health systems strengthening in Northern Togo
Source: PLoS One. 2017 Mar 16;12(3):e0173445. doi: 10.1371/journal.pone.0173445 (PMC5354273; doi:10.1371/journal.pone.0173445)
Supplement: S1 Table — (DOCX) [file pone.0173445.s001.docx]

**Supplemental Table 1**

| Supplemental Table 1. Participation enrollment by study site, HTH population-representative household survey of 4 catchment areas in Kara region, Togo, 2015 | | | | | |
| --- | --- | --- | --- | --- | --- |
|  | Adabawere | Kpindi | Sarakawa | Djamdé | Total |
|  | N, % | N, % | N, % | N, % | N, % |
| Census population (2010) | 6923 | 4935 | 3899 | 5700 | 21457 |
| Selected households | 719 | 393 | 414 | 396 | 1992 |
| Completed | 521 (73%) | 238 (61%) | 280 (68%) | 292 (74%) | 1331 (69%) |
| *No eligible member home* | 176 (25%) | 141 (36%) | 125 (30%) | 101 (26%) | 543 (28%) |
| *Refused* | 16 (2%) | 11 (3%) | 5 (1%) | 2 (1%) | 34 (2%) |
| *Partially completed* | 2 (<1%) | 1 (<1%) | 1 (<1%) | 1 (<1%) | 5 (<1%) |
| *Incapacitated/ other* | 4 (<1%) | 2 (1%) | 3(1%) |  | 9 (<1%) |
| Live birth in past 10 years | 369 | 209 | 243 | 254 | 1075 |
| Live birth in past 2 years | 162 | 98 | 111 | 119 | 490 |
